# Combining GPS, GIS, and accelerometry to explore the physical activity and environment relationship in children and young people – a review

**DOI:** 10.1186/s12966-014-0093-0

**Published:** 2014-09-13

**Authors:** Paul RW McCrorie, Candida Fenton, Anne Ellaway

**Affiliations:** MRC/CSO Social and Public Health Sciences Unit, University of Glasgow, 200 Renfield Street, Glasgow, G2 3QB Scotland

**Keywords:** Physical activity, Global Positioning System (GPS), Geographic Information System (GIS), Accelerometer, Environment, Children, Young people

## Abstract

The environment has long been associated with physical activity engagement, and recent developments in technology have resulted in the ability to objectively quantify activity behaviours and activity context. This paper reviews studies that have combined Global Positioning Systems (GPS), Geographic Information Systems (GIS) and accelerometry to investigate the PA-environment relationship in children and young people (5–18 years old). Literature searches of the following bibliographic databases were undertaken: Sportdiscus, Medline, Embase, CINAHL, Psychinfo and Applied Social Sciences Index and Abstracts (ASSIA). Fourteen studies met the inclusion criteria, and covered topics including greenspace use, general land use, active travel, and the built environment. Studies were largely cross-sectional and took place across developed countries (UK, USA, Canada, New Zealand, and Australia). Findings suggest that roads and streets, school grounds, and the home location are important locations for total PA, and moderate to vigorous PA (MVPA). The relationship between greenspace was positive, however, multiple definitions and outcome measures add complexity to the results. MVPA was more likely in those exposed to higher levels of greenspace compared to sedentary individuals. Total MVPA time in greenspace is low, but when framed as a proportion of the total can be quite high. Domestic gardens may be an important area for higher intensity activity.

Researchers are encouraged to show transparency in their methods. As a relatively new area of research, with ever-evolving technology, future work is best placed in developing novel, but robust, methods to investigate the PA and environment relationship. Further descriptive work is encouraged to build on a small but increasing knowledge base; however, longitudinal studies incorporating seasonal/weather variation would also be extremely beneficial to elicit some of the nuances associated with land use. A greater understanding of geographic variation (i.e. within and between countries), as well as urban/suburban and rural dwelling is welcomed, and future work should also include the investigation of psycho-social health as an outcome, as well as differences in socio-economic status, sex and adiposity.

**Electronic supplementary material:**

The online version of this article (doi:10.1186/s12966-014-0093-0) contains supplementary material, which is available to authorized users.

## Introduction

Physical activity (PA) is an important contributor to physical and mental health in young people [1-3]. The determinants of PA are multiple and inter-related, and ecological models suggest that many levels of determinants interact to influence domain specific behaviour [4]. One level of interest is the physical environment, and the number of studies exploring environmental correlates/determinants of PA has grown rapidly in recent years [5-11]. Some have investigated individual environmental determinants such as parks, recreation and greenspace [12,13]; some have concentrated on particular domains of PA such as active travel [14]; and others have focussed on the measurement of environmental exposure, including, questionnaires [15] and Global Positioning System (GPS) devices [16]. To date, no paper has summarised the research that uses objective measures of environmental exposure and both PA level and its context, in children and young people.

Part of the recent growth in the environmental determinants of PA literature can be attributed to the advancement of technology; improvements in the measurement of PA (e.g. accelerometry) provide a more accurate representation of this health behaviour, and advances in computer software (e.g. Geographic Information Systems; GIS) provides the tools to measure physical environmental characteristics of the land that people inhabit. Moreover, it is now possible to provide context to PA with the advent of portable, consumer-level Geographic Positioning System (GPS) devices. Previously, researchers would often measure the effects of the environment through questionnaire or self-report [15,17-19]. The outcomes have therefore been based on perceptions of the environment, as well as perceptions of activity levels - a potential same source bias concern [20]. The heterogeneity of design and methodology, particularly in PA and environment measurement, has led to inconsistencies in some hypothesised relationships between the environment and PA [11], including greenspace [13]. As a theoretically valuable resource for PA [21], one would hypothesise that having greater access to green spaces would result in greater levels of PA, however, according to a recent review [13] the relationship is still unclear. Approximately 40% of the studies reviewed (n = 50) found a positive relationship between greenspace and PA, six of which were for children/teenagers. Conversely, 15 studies found no association between greenspace and PA, and 13 were weak or of mixed outcome. A further two found a negative relationship between the two variables. Of the 50 studies included, the majority (n = 41) used self-report methods of PA assessment - only nine used accelerometry. Moreover, GIS based environmental exposure has had the tendency to use a mathematically derived buffer surrounding individuals’ residential location to define the accessibility of nearby greenspace to the participant. One reason for the inconsistencies from this type of approach is that proximity to greenspace may not translate to actual use, and Lachowycz and Jones [13] suggested that more research is required to investigate if, and how, greenspace is actually experienced. The advent of GPS devices allows for the where and when of PA to be investigated [8], and it has been suggested [6] that the combination of GPS, GIS, and accelerometry data would be a useful addition to the current understanding using self-reported measures. It has also been cited that environmental correlates of PA in youth tend to be more credible when results have been based on objectively measured environmental variables [11]. However, being novel in approach, there is also a tendency to use these technologies (e.g. GPS) without being fully aware of their capacity, and/or limitations. A current example of this may be the potential bias associated with ‘selective daily mobility’ [22], whereby spurious causal inferences between environmental factors and health behaviours are evidenced, as a result of GPS derived outcomes, but are actually confounded by other, unmeasured, factors (e.g. intrapersonal variables such as positive attitudes, and high self-efficacy). According to Chaix and colleagues, “this bias stems from the fact that measures of accessibility to given environmental resources are also determined from the locations that were specifically visited to use the corresponding resources” [22, p.48]. With this type of research being in its relative infancy, a review of the current literature - using these technologies within a specific population - may be beneficial for further work in the area, particularly as the authors are unaware of any paper that has attempted to summarise the research that has used objective approaches to both physical activity levels and its context, in addition to any potential environmental correlates.

The purpose of this review was to (i) synthesise and summarise the research that has used the combination of GPS, GIS, and accelerometry to investigate the physical environment/PA relationship among young people (5 – 18 years old) and (ii) identify gaps in knowledge that future research should address.

## Methods

### Search strategy

The initial stage was to establish the three core concepts of the paper: 1) physical activity, 2) movement monitoring (accelerometry) and 3) mapping (GPS and GIS). Following careful consideration of potential databases, the following were used for the main search: Sportdiscus, Medline, Embase, CINAHL, Psychinfo and Applied Social Sciences Index and Abstracts (ASSIA).

Terms sets were developed for each concept, using both free text and index terms. For the concepts of movement monitoring and mapping, term lists included the proper and trade names of devices and technologies (e.g. Actigraph, Garmin). Trial searches were then run to ensure that they retrieved all key papers, and terms lists were revised to improve precision and recall. Final searches were run on the 12th of June 2013 and all references were exported to bibliographic software (Endnote) and de-duplicated.

### Eligibility

Our review included studies which were not limited in methodological design, or quality. The following criteria were used to assess eligibility: (i) The study used accelerometers to measure PA in humans; (ii) the study used GPS devices to measure context of PA behaviour; (iii) the study combined the accelerometer and GPS data for use in a GIS package; (iv) the population investigated were children and young people between the ages of 5 and 18 years old.

### Selection

The lead author screened all articles and subsequently narrowed the search results based on eligibility. Initially, all returned articles were screened based on titles. All papers with clear deviation from the review topic were discarded. Secondly, abstracts of remaining articles were screened, and either passed on to the next stage for full text retrieval, or removed due to clear irrelevance and non-fulfilment of eligibility criteria. The third step retrieved the full text of remaining articles. Each paper was assessed against the eligibility criteria and included or excluded. The reference lists of all included papers were screened for further relevant papers.

### Data extraction

The data extracted included: general characteristics (first author, year, location, and journal); design and sample population; measurement devices used and accompanying details; GIS information, including the environment/neighbourhood variables measured; results and findings.

#### Findings

**Figure 1:**
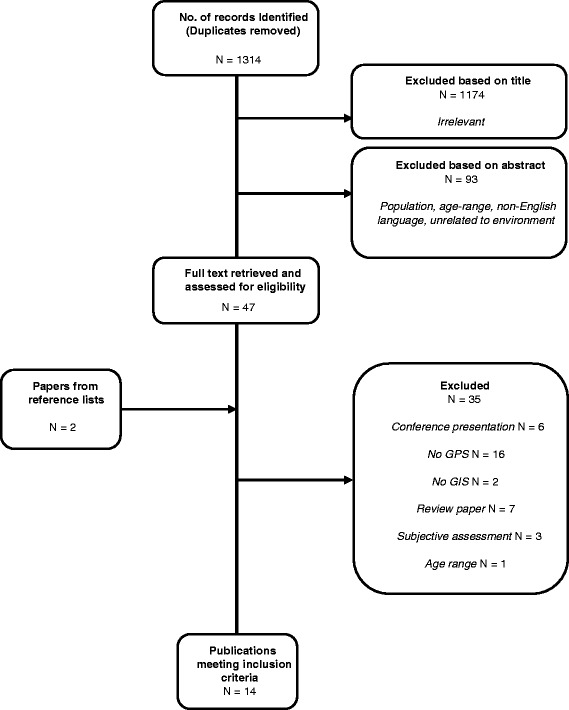
**Flow chart of the selection and exclusion process of articles.**

The flow chart representing study selection, including reasons for exclusion, is summarised in Figure 1. Following removal of duplicates, the literature search yielded 1314 articles. From this initial pool, 1174 were excluded based on irrelevant titles, and a further 93 removed upon reading the abstract content. The wrong population group, age ranges, and unrelated subject matter (i.e. not related to environmental determinants) were primary reasons for removal. Full text was retrieved for 47 articles and 12 met the eligibility criteria. Two papers were found upon screening of the reference lists of eligible papers, resulting in 14 papers in total.

### Characteristics of included papers

Of the 14 papers included, seven were conducted in the UK [23-29], four in the US [30-33], two in New Zealand [34,35], and one in Canada [36] (Table 1). With a subject topic that includes PA, health, geospatial, environment, and place based elements, the sources for publication were varied. The majority (92%) of the studies were cross-sectional analyses, with one involving a longitudinal approach [33]. Multiple papers included in this review were focused analyses of the same, but larger, projects, including: the Sport, Physical Activity and Eating Behaviour: Environmental Determinants of Young People project (SPEEDY; UK [23,25]); the Personal and Environmental Associations with Children’s Health project (PEACH; UK [24,26,28,29]); Healthy Places; the Trial of Activity for Adolescent Girls project (TAAG 1 & 2; US [32,33]); the ICAN project (NZ [34]); and the Children’s Activity in their Local Environment study (CALE; Canada [36]). The ages of those included ranged from 5–18 years old, with the 10–12 year old age-group being the focus of five papers. Sample sizes varied greatly (Mdn = 119, range = 24–1,053) and depended on the research question being investigated (e.g. validation work or descriptive studies). Two of the largest sample sizes (n = 902, 1053) were from one large scale study - the PEACH project.

**Table 1:** **General characteristics of included articles**

**First author [reference]**	**Year**	**Journal**	**Location/design**	**Sample size, % male**	**Age (yrs)**	**PA measurement**	**GPS measurement**
Coombes, E. [23]	2013	Health and Place	Norfolk, UK		9-10	Actigraph GT1M	**Garmin Forerunner 205**
			• Cross-sectional	• n = 100			
			• Part of a larger project – **SPEEDY**	• 47% Male			
Almanza, E. [30]	2012	Health and Place	California, USA		8-14	Actigraph GT2M	**Globalsat BT-335**
			• Cross-sectional	• n = 208			
			• Part of a larger project - **HEALTHY PLACES**	• 48% Male			
Lachowycz, K. [26]	2012	Health and Place	Bristol, UK		11-12	Actigraph GT1M	**Garmin Foretrex 201**
			• Cross-sectional	• n = 902			
			• Part of a larger project – **PEACH**	• 47% Male			
Oreskovic, N. [31]	2012	Geospatial Health	Revere, Massachusetts, USA	• n = 24	11-12	Actigraph GT1M	**Garmin Forerunner 201**
			• Cross-sectional	• 42% Male			
Rainham, D. [36]	2012	American Journal of Preventive Medicine	Halifax, Nova Scotia, Canada	• n = 316	12-16	Actigraph GT1M	**Globalsat EM-408 SiRF III receiver**
			• Cross-sectional	• 53% Male			
Rodriguez, D. [33]	2012	Health & Place	San Diego, CA Minneapolis/St. Paul, MN, USA	• n = 293	15-18	Actigraph 7164	**Garmin Foretrex 201**
			• Longitudinal study – **TAGG** study participants	• 100% Female			
Rodriguez, D. [32]	2012	Journal of Physical Activity and Health	San Diego, CA Minneapolis/St Paul, MN, USA		15-16	Actigraph 7164	**Garmin Foretrex 201**
			• Cross-sectional	• n = 42			
			• Data collected as part of - **TAAG2** study	• 100% Female			
Southward, E. [28]	2012	American Journal of Preventive Medicine	England, UK		11-12	Actigraph GT1M	**Garmin Foretrex 201**
			• Cross-sectional	• n = 84			
			• Part of a larger project – **PEACH**	• not stated			
Cooper, A. [24]	2010	American Journal of Preventive Medicine	London, UK		10-11	Actigraph GT1M	**Garmin Foretrex 201**
			• Cross-sectional	• n = 137			
			• Part of a larger project – **PEACH**	• 44% Male			
Maddison, R. [34]	2010	Pediatric Exercise Science	Auckland, NZ		12-18	Actigraph 7164	**Garmin Forerunner 305**
			• Cross-sectional	• n = 79			
			• Part of a larger project – **ICAN** study	• 58% Male			
Wheeler, B. [29]	2010	Preventive Medicine	Bristol, UK		10-11	Actigraph GT1M	**Garmin Foretrex 201**
			• Cross-sectional	• n = 1053			
			• Pilot of a larger project – **PEACH**	• 47% Male			
Quigg, R. [35]	2010	Preventive Medicine	Dunedin, NZ	• n = 176	5-10	Actigraph GT1M	**Globalsat DG-100**
			• Baseline data from a 2-year intervention study - **CALE**	• 48% Male			
Jones, A. [25]	2009	International Journal of Behavioral Nutrition and Physical Activity	Norfolk, UK		9-10	Actigraph GT1M	**Garmin Forerunner 205**
			• Cross-sectional	• n = 100			
			• Part of a larger project – **SPEEDY**	• 47% Male			
Mackett, R. [27]	2007	Built Environment	Hertfordshire/Lewisham, UK	• n = 82	8-11	RT3 tri-axial accelerometer	**Garmin Foretrex 201**
			• Cross-sectional	• 43% Male			

### PA and GPS instruments used

The most widely used accelerometer was the Actigraph GT1M (included in 9/14 studies; Actigraph, LLC., Pensacola, FL), followed by the Actigraph 7164 (n = 3) and GT2M (n = 1) devices (Table 1). The RT3 tri-axial accelerometer (Stayhealthy, Inc., Monrovia, CA) was the other device represented. Garmin devices (Garmin International, Inc., Olathe, KS) were the most popular GPS monitoring tool (n = 11/14 studies), with the Garmin Foretrex 201 (n = 7), Forerunner 201 (n = 1), Forerunner 205 (n = 2), and Forerunner 305 (n = 1) all used. The remaining GPS devices were the Globalsat BT-335 (n = 1), DG-100 (n = 1), and the EM-408 receiver (n = 1) (GlobalSat Worldcom Co., New Taipei City, Taiwan).

### Study topics

(Additional file 1: Table S2) identifies the detailed data characteristics of each included study. Four studies [26,29,30,35] investigated the contribution/association of green spaces and PA; five studies [23,25,31,34,36] investigated general land use exposure, using the monitoring devices to assign land use categories and intensity classifications to GPS points. Three studies [24,27,28] investigated active travelling/walking patterns, using the GPS technology to map walking trips, and the walk/drive to school. A further article [33] investigated the effects of the built environment, around each GPS point, and the odds of the GPS points being of higher intensity activity. The final study [32] used the combination of devices and technology to investigate the agreement between diary-listed locations and those objectively measured. The results of this study indicated that between 86% and 100% of all diary-reported locations matched that of the GPS identified locations. All other results, sub-categorised by the main environmental interest of the article, are presented in the following sections (Additional file 2: Table S3 for summary of findings).

### Study findings

#### Greenspace

Four studies investigated the use/exposure of greenspaces and PA. Each study used differing methodologies and differing definitions of what was considered greenspace. Two papers were cross-sectional analyses of the same larger longitudinal project – the PEACH project [26,29], with analyses one year apart.

Two studies [29,30] used logistic regression modelling to produce odds ratios. In the first of these [30], greenspace was measured by a novel index that categorised each GPS point by its level of greenness in two different communities: a conventional community design; and a modified design aimed at encouraging PA. The greenness of each GPS point was associated with the likelihood of each GPS point being of moderate-vigorous intensity (compared to sedentary) and identified odds ratios of 1.34 and 1.39 (conventional and modified community, respectively). Being exposed to greenness for more than 20 mins per day resulted in 4.72 times the daily rate of MVPA compared to those with nearly zero daily exposure. The second study [29] was UK based, and investigated the association of data-points falling on greenspace and the likelihood of those data-points being moderate-to- vigorous intensity. Exposure to greenspace resulted in an odds ratio of 5.77 (p < 0.01; compared to indoors) for boys and 5.12 (p < 0.01) for girls. Being outdoors and in greenspace was associated with greater odds of MVPA than being outdoors and in non-greenspace – 1.37 for boys and 1.08 for girls. Only 13% of all activity was spent outdoors, with 8.6% and 6.1% of all MVPA occurring in greenspace (boys and girls, respectively).

The remaining two studies [26,35] investigated the contribution of greenspace use to the PA levels of their respective samples. Lachowycz and colleagues [26] found that 2.4 mins/day of MVPA occurred in greenspace during weekdays and 3.5 mins/day at the weekend. As a percentage of total MVPA, the weekday figure constituted 4.8% and the weekend figure 9.1%. Seasonal differences were found for the percentage of total time spent in MVPA occurring in parks during weekdays; percentage time was lower in winter (7.0%) and spring (7.7%), compared to summer (17.2%) and autumn (11.2%) (p < 0.001). The final paper [35], identified that 1.9% of total daily PA (TDPA) was located in a city park with a playground. Higher proportions of TDPA was found in the obese (2.7%) compared to the normal (2.0%) and overweight (1.1%) participants (p = 0.023). Boys spent more time in city parks with playgrounds than girls (2.4% vs 1.5%, p = 0.036). Very little activity occurred in city parks after 3pm (0.5%), however, this was significantly different across age groups (Additional file 1: Table S2).

#### General land use

Five studies [23,25,31,34,36] investigated the contribution of differing land uses on young people’s PA. Two papers were individual analyses of the SPEEDY project [23,25]. The analysis conducted by Jones and colleagues [25] included the comparison of bout MVPA carried out indoors and outdoors, inside and outside the neighbourhood (800 m pedestrian buffer network around residence), and in specific land use types. More time in MVPA was accumulated (45 mins/day vs 28 mins/day, p = 0.002) for those participants who spent more time outdoors, a difference consistent across urban and rural dwelling status and gender. 63% of all MVPA bouts occurred inside participant’s neighbourhoods. The mean length of time spent in bouts of MVPA was significantly longer in boys, both inside, and outside, their neighbourhoods (p < 0.05); however a higher proportion of total MVPA was found in girls, inside their neighbourhoods (67% vs 60.4%). The mean length of time spent in bouts of MVPA inside and outside the neighbourhood was dependent on urban and rural dwelling; urban children were significantly more active in MVPA bouts inside their neighbourhoods (26 mins vs 23 mins, p < 0.05), and rural participants spent more time in MVPA bouts outside their neighbourhoods (17.3 mins vs 13.8 mins, p < 0.05). The second SPEEDY paper [23] calculated the percentage of recorded time that children spent undertaking light, moderate, and vigorous activity, in addition to bout and non-bout MVPA, in nine different land use categories. A greater proportion of recorded light intensity time (24%) compared to moderate (20%) and vigorous (18%) was spent in buildings (p < 0.001). This relationship was also evident for roads and pavements (13% vs 12% vs 9%, p < 0.001; light, moderate, and vigorous, respectively). A greater percentage of vigorous activity (30.6%) occurred in domestic gardens compared to light (28.6%) and moderate (26.8%) PA (p = 0.009). Similar patterns were evident for parks (p = 0.011) and grassland (p = 0.005). The authors emphasise that the absolute number of minutes spent in vigorous activity in each of the different land categories was low (<1.5 min/day), although they suggested that green environments may be supportive of higher intensity activity. A significantly greater percentage of bout MVPA (17%) compared to non-bout MVPA (9.1%) was found in roads and pavements (p < 0.001). The reverse relationship was found for MVPA occurring in buildings (21.5 vs 6.9%; p < 0.001, non-bout v bout MVPA), other built land uses (15.7% vs 10.6%, p < 0.015), and domestic gardens (29.2% vs 20.6%, p < 0.001), where non-bout MVPA was greater than bout-MVPA.

Three other studies investigated the contribution of land use categories to young people’s PA. Oreskovic and colleagues [31] reported that 29% of winter MVPA was spent in land use categorised as streets/walking, second to the 43% spent at home. In spring, streets/walking contributed 44% of total MVPA. During the summer, the largest contributor to MVPA was parks/playgrounds, contributing 57% of all MVPA. Rainham and colleagues [36] investigated the total recorded time in MVPA, in different locations, across urban, suburban and rural locations in Nova Scotia, Canada. Across all levels of dwelling categories, the percentage of total MVPA time was accumulated substantially across the home, school and commuting locations (Additional file 1: Table S2). Although small in terms of percentage of the total, MVPA in greenspace was greater in rural children (girls = 4.8%, boys = 5.6%) compared to suburban (girls = 2.5%, boys = 3.9%), and urban (girls = 1.3%, boys = 0.6%) participants. Moreover, mean time spent in MVPA was attributed to four different locations (home, school, commuting, and other) by urbanicity and SES (low and high; low defined as < $50,000 household income). No differences were found in mean MVPA time at *home*, across the levels of urbanicity, or SES. Urban participant’s mean MVPA (45.7 mins; total recorded MVPA) at *school* was greater than suburban (18.6 mins) and rural participants (29.8 mins) (p < 0.001), a relationship also evident in the *commuting* category, (110.3 vs 31.5 vs 19.5 mins; p < 0.001) and the *other* category (19.7 vs 14.8 vs 12.0 mins, p = 0.03). No differences were found by SES across the urbanicity categories.

The final study was conducted in New Zealand [34] and focused on describing the location and intensity of free-living bout, and non-bout, MVPA at school (1 km Euclidean buffer surrounding school) and inside the home environment (150 m Euclidean buffer around residence) during the week and at weekends. During weekdays, a total of 510 hours (across four days) was spent inside the 1 km buffer of school (65 hours during school time), 14% of which was accumulated MVPA. A total of 268 hours was spent inside the 150 metre buffer surrounding the home location, 38 hours of which was spent in MVPA. During the weekend, 55 hours was spent inside the school buffer, 11% of which was spent in MVPA; 30 hours was spent inside the home environment during the weekend, 12% of which was MVPA.

#### Walking/active travelling

Three studies investigated active travelling or walking trips [24,27,28]. Two papers [24,28] originated from the PEACH project, the earlier of which [24] investigated the contribution of the school journey to PA and MVPA in the hour before school in a group of 10/11 year olds. Matched data were separated into “journey” and “playground” and activity levels were found to be higher in the journey component (2131 cpm) compared to the playground (1089 cpm, p < 0.001). Approximately half of the matched GPS points were evident during the journey, of which 1.6 mins were MVPA. Only 0.6 mins of MVPA were recorded in the playground. The second paper from the PEACH project [28] investigated the contribution of the school journey to daily PA when the cohort were 11/12 years old. Adding to the earlier analysis by Cooper and colleagues, both the before and after school periods were recorded and analysed. Approximately 50% of the journey to and from school was MVPA. No differences were found between MVPA times in each journey. There were no differences between boys and girls PA and the journey to and from school; however, the proportion of total daily MVPA in the school journey was higher in girls than in boys (35.6% vs 31.3%, girls and boys, respectively). A significant positive linear relationship was found between distance walked from the school journey and overall daily MVPA.

The final paper [27] was one of the first to combine accelerometry, GPS, and GIS in young people. Using the outcome variables of speed, intensity (energy expenditure), and angle (straighter patterns indicating more structured behaviour than multiple changes in angles), in combination with all GPS points falling within the land use category of ‘Roads, tracks and paths’ or ‘other space’, Mackett and colleagues found that children walked faster (p < 0.001) and straighter (p < 0.001) when accompanied by an adult. Additionally, boys walked faster (p = 0.013), expended more energy (p < 0.001), and walked in a straighter fashion (p = 0.012). Overall, the participants walked faster (p < 0.001), straighter (p = 0.019), and more intensely (p < 0.001) on roads compared to open space.

#### Built environment characteristics

Rodriguez and colleagues [33] investigated the momentary association of built environment characteristics and simultaneous PA and sedentary behaviour in adolescent females across two different American cities (San Diego and Minneapolis). Fifty metre buffers encircled each individual GPS point, and a collection of built environmental characteristics were associated with the PA intensity classification of each GPS point. In San Diego and Minneapolis, GPS points that occurred in areas with higher population density (OR = 1.01 and 1.04, San Diego and Minneapolis, respectively), and with schools present (OR = 1.69 and 2.14), were more likely to have MVPA (ref group sedentary). In Minneapolis alone, the presence of parks resulted in higher odds of MVPA (OR = 1.86). The odds of MVPA at the weekend were lower in both cities (OR = 0.39 and 0.62); and both longer road length (OR = 0.38 and 0.43) and number of food outlets (OR = 0.73 and 0.71) were also associated with lower odds of MVPA.

## Discussion

### Study characteristics

The purpose of this review was to scope the literature for articles that had combined accelerometry, GPS and GIS into place-based research of children and young people. Viewed as an important development in the field of environmental determinants of physical activity, many researchers have called for the inclusion of this type of objective approach, therefore enabling the investigation of PA context, whilst complimenting existing work that has confined their analyses to the association of contextual neighbourhood characteristics (usually through buffers) and PA [9,11,13,37]. As this type of research is in its formative stages, our review has attempted to synthesise and summarise the current literature from a methodological perspective, and research findings context.

Conducted across ‘developed’ countries, including the US, UK, New Zealand, and Canada, the research using this type of approach, to date, has primarily focused on the description of participant’s exposure to different environmental characteristics, in addition to the identification of certain characteristics conducive to specific PA intensities (predominately MVPA). Many of the included articles within this review are part of larger studies, where multiple research questions have been investigated [23,25], or similar questions answered at multiple ages of the sample [24,28]. Delineating outcomes of the included studies by age was not presented in our review however the 10–12 year age group was popular. This age group represents the transition stage where children move from lower/primary to upper/secondary school and is viewed as an important age for PA levels; as adolescents move through adolescence, activity levels begin to decline, particularly for girls, and never recover into adulthood [38]. The change in social and physical environment associated with the move from lower to upper school is therefore an important period for investigation and the development of interventions.

### Data integration and manipulation

There is no standard method of interpreting GPS data [9,36]. The studies in this review used different methods to integrate GPS and PA data [e.g. 25,26,31], and all had different exclusion and inclusion criteria for the GPS and/or PA data. In some instances, these criterion were less defined than others [23,25], a reflection of the type of research question being answered. As is common in ‘count’ based activity monitoring research, a certain number of active hours are required per day before a day can be considered valid for analysis, taking into consideration non-wear time. Additionally, a certain number of valid days are required per participant before being included. This is generally a reflection of capturing enough days to calculate what an average PA day would look like; the more days included, the more likely a mean score would represent the data accurately. This type of approach could be considered critical if the research question is directed towards PA prevalence, i.e. interpreting the number of participants within a sample that meet current recommended daily guidelines. However, as is the case with some GPS/PA research, this may not be the primary focus, and the concern lies more with exploring the PA context, and associated intensity classification/energy expenditure, to identify environmental characteristics that are conducive to PA, with the ultimate goal of informing policy (e.g. transportation, new housing communities, and PA facility distribution). Consequently, the outcome variables within the included studies were all quite different, and some were not explicit with how they were calculated. For instance, Rainham and colleagues [36] presented many of their results as the percentage of total hours in MVPA per environmental location. However, it was not clear how MVPA was derived, or if they used any inclusion/exclusion criteria for their PA and GPS data. When including participants with differing numbers of days, it would seem appropriate to use a proportion of total MVPA time (in this case, total time across the whole recorded data) to represent the data in a standardised format. In contrast, Jones and colleagues [25] explicitly stated that accelerometer wear time was not important, that their MVPA bouts were based on specific threshold classifications, and that many of their outcomes were means per child across four study days. Much more can be done by authors, for the sake of study replication, to be explicit and transparent in their integration and analysis techniques.

The nuanced methodological approaches of the included studies render the interpretation of research findings difficult. For instance, multiple definitions have been used to account for green spaces: Almanza and colleagues [30] used a greenness index, which placed a value of greenness onto a 30-metre pixel resolution map; Lachowycz et al. [26] used national and local digital maps to classify greenspace into categories such as ‘private gardens’, ‘parks’, and ‘school grounds’; and Quigg et al. [35], focused their investigation on city parks which contained play areas for children. Different definitions may require different data sources, which could lead to under or over-reported relationships. Furthermore, studies deal with the inherent errors associated with GPS accuracy, particularly in and around buildings (i.e. signal multipath errors), differently. Rodriguez et al. [33] removed all datapoints falling within a 60 metre buffer of school and home; Oreskovic et al. [31] included all points falling within 25 metres (although increased for some participants) of the geocoded home location, and 100 metres of school, classifying them as ‘home’ and ‘school’, respectively; Almanza et al. [30] identified a point as being at home if it fell within a 30 metre Euclidean buffer around the geocoded home address; and Maddison et al. [34] included a 150 metre buffer around the home location to account for GPS error, and include all home-based activity. Other studies excluded data if they fell outside the mapping capabilities of their data sources [26,29].

A further variation between papers related to missing and inconsistent GPS data. Overall, very few articles made mention of how they worked with these common problems. Some identified and geographically re-categorised irregular data to a known location [36]; some imputed GPS points using specific formulae [34]; others automatically categorised accelerometer points with no GPS equivalent as points lying indoors [24,26,29]. Some articles discarded all data that were unmatched [31,33], meaning that all GPS points with no accelerometer equivalent and accelerometer epochs with no GPS equivalent were removed from analysis. Some papers were explicit in the removal of certain extreme/outlier data in their GPS data: Almanza and colleagues [30] removed all GPS points that had associated speeds of 105 mph (>169 kph), and Quigg et al. [35] removed all points that had elevation heights of >800 m, reflecting the topography of Dunedin (sea level - 739 m). Ultimately, there does not seem to be a consensus in place that proposes guidance for dealing with these types of data issues. This will come with time, as it has with PA monitoring work, but many of the problems relate to the infancy of the research. Not enough is known about the optimal methods to approach data manipulation, and time is needed to find the answers to these questions.

### Research findings

Although difficult to synthesise, some patterns arose from the papers included in this review. Roads and streets, school grounds, and the home location are important locations for total PA, and MVPA [31,34,36]. In the study conducted by Rainham and colleagues [36], urban children accumulated greater than 55% of their total MVPA whilst commuting to and from school, and other locations (e.g. the mall or restaurants). Oreskovic and colleagues [31] found that between 11% (summer) and 30% (winter) of total MVPA was accumulated whilst on the streets/walking. Additionally, Coombes and colleagues [23] identified 13% of light, 12% of moderate, and 9% of vigorous activity occurred on roads and pavements (sidewalks). Articles focusing on active travel support these data by identifying that each journey to and from school contributed, on average, between 32-36% of total daily MVPA, increasing linearly as distance from school increased [28]. The authors concluded that strategies to maintain or increase active travel to school may be an important public health approach that tackles the decline in PA levels seen throughout adolescence. Park and stride campaigns have been implemented in the UK (http://www.livingstreets.org.uk), whereby car exclusion zones are implemented around schools, meaning parents have to drop their children at a pre-determined distance from school (e.g. a 5 or 10 minute walk). This type of policy implementation may have the potential to increase overall PA and MVPA levels whilst positively impacting other important health related outcomes such as road safety, and car exhaust emissions.

Mixed results were found in those articles investigating greenspace use and PA levels. This finding is in line with previous reviews that have synthesised the association between greenspace and PA [13]. Commonalities between studies included the general consensus that greenspace use, as an absolute measure of time, was low. For example, Lachowycz et al. [26] reported that MVPA time spent in greenspace per day was as low as 2.4 mins on weekdays, and 3.5 mins at weekends. Rainham and colleagues [36] also found low levels of MVPA attributed to greenspace, a finding that increased as level of urbanicity (0.8 mins for boys, 2.2 mins for girls) lessened and moved toward rurality (4.9 mins for boys, 3.8 mins for girls). Moreover, Quigg and colleagues [35] reported that only 1.9% of total daily PA was located within a city park with a playground. Contrasted with these results are those by Jones et al. [25] and Coombes et al. [23] who identified gardens to be amongst the most exposed land use. Rural participants were more likely to use farmland and grassland than urban children, a finding more consistent with boys than girls [25]. Coombes and colleagues [23] also suggested that greenspace may be more supportive of vigorous intensity PA, with 31% of all vigorous activity occurring in domestic gardens. A point not mentioned by the authors was the significant use of greenspace that was of light intensity, with 26 mins/day (29% of total light intensity) being accumulated in domestic gardens (compared to 4 mins of vigorous and 7 mins of moderate). The benefits of light intensity activity should not be underestimated, as this type of activity has been shown to have significant physiological benefits [39]. Of the two articles [29,30] that produced odds ratios relating MVPA and greenspace, both suggested that the exposure to greenspace and odds of MVPA were greater compared to sedentary [30], and to MVPA indoors, and outdoor non-greenspace use [29].

The mixed results that seem to accompany the greenspace literature may lie with the intricacies of the research questions, greenspace definitions used, the study population, and the study area (e.g. country, and urban/rural). Some articles presented in this review highlighted urban/rural differences for instance (e.g. [25,36]), and seasonal variation may also play an important role in greenspace use [26,31]. Both should be considered in the design of future work. Ultimately, the heterogeneity across multiple conditions/variables makes it difficult to detect the efficacy of greenspace to foster PA levels. Studies that are equivalent in their methodologies, and are comparable geographically and topographically, will be extremely useful. For example, in the UK, the SPEEDY, PEACH, and a newly launched project named SPACES (Studying Physical Activity in Children’s Environments across Scotland), share many of their methodologies. The effects of greenspace are not necessarily restricted to physical activity or indeed ‘physical’ outcomes in general. Green spaces can offer aesthetically pleasing areas that impact psycho-social outcomes [40,41]; outcomes that activity monitoring devices are unable to detect but may be equally important for overall health and wellbeing.

### Future directions

The introduction and integration of accelerometry and GPS data is a relatively new development in the field of environmental determinants of PA research. Many opportunities exist in the development of the devices themselves, with device manufacturers developing better technology as the research field progresses and researcher requirements become more advanced. In relation to GPS, common issues such as satellite acquisition time and better discrimination of indoor activity are already taking place. Many of the GPS devices included in this review would almost certainly be classified as obsolete. Newer devices (e.g. Qstarz, Qstarz International Ltd, Inc, Taipei, Taiwan) are able to use the quality of the satellite signal alongside the associated noise to discriminate indoor activity. The articles included from the UK PEACH project for instance [24,26,29], automatically defaulted accelerometer points with no GPS equivalent as ‘indoors’. With newer technology, better, more informed decisions can be made, resulting in fewer misclassifications and more accurate findings.

Computing processing power and memory capacity are becoming increasingly important to the development of this type of research. GPS devices are being made with larger storage capacities to enable higher frequency data capture, and when combined with high frequency activity monitoring data, both of which have been recording for upwards of seven days, 10 hours per day, the resulting files become sizeable. When larger, population level studies are considered, one question that needs to be answered is whether the computing power is present to cope with the huge requirements of running spatial queries on tens of millions of data points.

The advancement of GPS research can also take place by eliminating error within the data itself. The elimination of ‘bad’ data is of significant importance and areas such as cluster detection and mode of transport identification [42], in addition to trip/journey detection [43,44], are all methods that can be used to reduce error in the data as well as increase the quality of the context from which the PA derives. Within GIS focused research, work can, and is, being conducted which measures the ‘neighbourhood’ component of exposure more accurately, and GPS technology will complement and assist the advances in this field. The authors point the reader in the direction of work by Rainham and colleagues [37], and Schipperijn and colleagues [45] that addresses standard deviational ellipses, minimum convex polygons, and kernel density estimation analyses within GIS.

In contrast to the ‘neighbourhood’ approach, one of the major benefits of GPS is the ability to capture daily mobility, and in particular, multi-place activity. Chaix and colleagues [22,46], have previously stated that there is a growing recognition that most people only spend a limited amount of time each day within their residential neighbourhoods. With the addition of GPS, there is the potential to gain a more comprehensive understanding of the environment/health-behaviour relationship. However, to demonstrate this type of relationship accurately, it is important to understand the potential biases associated with using the results of GPS derived measures as they relate to environmental resources. Mentioned within the introduction, selective mobility bias occurs through the confounding effects of unmeasured variables (e.g. intrapersonal factors) that are also related to the health-behaviour of interest. The potential bias arises when spatial queries, regarding accessibility to environmental resources, are run from locations (the very environmental resource predicted to affect health) visited specifically to engage in the health behaviour of interest. The resulting associations would suggest greater exposure to certain environmental resources, but would, in fact, be biased. According to Chaix and colleagues, at the very minimum, GPS data should be filtered to exclude these places, leaving only valid reference locations to assess accessibility to important environmental resources. To do so, one needs to understand more about where the activity is occurring (activity place), when the activity starts and stops, how the person arrived and departed from the activity place (trip detection), and importantly, the nature of the activity itself. Combining GPS, GIS, accelerometry, and mobility surveys (e.g. VERITAS, [46]) may reduce the bias associated with selective mobility, and therefore correct the estimated effects of the environment/health-behaviour relationship.

Other future directions available to researchers include the expansion of the physical activity construct when investigating environmental correlates. Most articles in this review concentrated on MVPA. This is not surprising considering the association of MVPA with health benefits [2], and the necessity to meet a certain level of this intensity of PA to meet national and international guidelines [47]. However, researchers should consider light intensity activity, in addition to sedentary behaviour, as current research suggests both have specific physiological pathways that act independently of each other on health [39,48].

The theoretical foundations of much of the work conducted in this area use an ecological approach [4], whereby multiple levels influence the behaviour of an individual. Future work can do well to integrate as many of these levels into the research questions/design as possible. Some of the work cited within this review successfully combined their GPS/GIS/accelerometry technical components with research questions that investigated not only physical environmental characteristics such as urban/rural classification [23,25,36], but also important considerations such as SES [36], obesity [29,35], gender [23,25,27,28,36], weekday and weekend patterns [26,34], and the physical seasons [26,31]. Attention should also be given to more specific environmental conditions such as weather and daylight length.

Although this review has been conducted with a quantitative focus, the incorporation of qualitative approaches that assists and supplements the technology-based section is also encouraged. Placed within a child/young person context, methods that incorporate walking interviews, place-based photography, and other creative methods with children and young people [49] will provide a more holistic understanding of the environmental determinants of children’s/young people’s PA.

### Strengths

This review, as far as the authors are aware, is the first to scope the literature for articles that have been conducted using GPS, GIS, and accelerometry in children and young people, and is therefore a timely body of work that will prove beneficial for academics venturing into this field. Although in its infancy, the synthesis of the current review will also inform those in the wider research sphere, such as funding bodies, local and national governing bodies, and policy makers alike.

A previous review [16] did include a small number of studies that have been included in the current review, however, the focus was specific to data loss within the GPS component, and not findings in the same sense as has been presented within this paper. The main reason for this review was to conduct an extensive systematic search of wide reaching literature databases to inform ourselves, and the research community, of the types of work that is being conducted within children and young people. With this as the primary aim, this review will provide researchers with an understanding of the current literature, as well as directions for potential future work.

### Limitations

A limitation of this review is that a small number of articles were included, primarily due to the narrowed focus of the subject topic. The authors are aware that certain elements of the inclusion criteria could be altered, even only slightly, and this would result in a greater number of potential papers being included. An example of this would be to broaden the physical activity monitoring component, allowing all objective monitoring (e.g. heart rate monitoring, and pedometers) to be included, and not just accelerometry [50,51]. Extending the review to the adult population would also increase the number of papers returned from any structured search [52].

As this paper was not a systematic review per se, multiple authors did not screen included papers, and data quality was not included. This introduces the possibility that some papers may have been excluded without debate, and those included may be of poor quality. Further limitations include the restriction of articles to the English language, and the absence of research outcomes by age category (e.g. young children, older children, adolescents and young adults). Consequently, results/ relationships between environmental characteristics and PA may be moderated by age.

## Conclusions

This review has attempted to scope and synthesise the current literature that has used GPS, GIS and accelerometry in children and young people. It is evident that the field is evolving quickly, however there is a risk that the research being conducted is non-replicable/repeatable due to study specific designs and, in some occasions, insufficient information on the procedures used. However, this review provides early indications that certain environmental characteristics, when measured objectively, have a significant effect upon the PA levels of children and young people. GPS data suggests that pavements (sidewalks)/roads, school grounds, and the home environment are particularly important. The significance of greenspace for PA behaviour, particularly in relation to more intense levels of PA is beginning to be observed. It is important that researchers continue to build upon the work conducted to date, using appropriate research designs and methodologies that investigate multiple variables, including social, psychological, and environmental levels. This will provide a strong evidence base that can inform policy development, with the aim to provide children and young people with the types of environment conducive to all types of informal and formal PA.

## Additional files

Additional file 1: Table S2. PA and place based variables/measures, and study findings of all included articles. Additional file 2: Table S3. Research questions and main findings of included articles.

## References

[1] Biddle SJ, Gorely T, Stensel DJ (2004). Health-enhancing physical activity and sedentary behaviour in children and adolescents. J Sports Sci.

[2] Strong WB, Malina RM, Blimkie CJ, Daniels SR, Dishman RK, Gutin B, Hergenroeder AC, Must A, Nixon PA, Pivarnik JM, Rowland T, Trost S, Trudeau F (2005). Evidence based physical activity for school-age youth. J Pediatr.

[3] Janssen I, Leblanc AG (2010). Systematic review of the health benefits of physical activity and fitness in school-aged children and youth. Int J Behav Nutr Phys Act.

[4] Sallis JF, Cervero RB, Ascher W, Henderson KA, Kraft MK, Kerr J (2006). An ecological approach to creating active living communities. Annu Rev Public Health.

[5] de Vet E, de Ridder DT, de Wit JB (2011). Environmental correlates of physical activity and dietary behaviours among young people: a systematic review of reviews. Obes Rev.

[6] Duncan MJ, Badland HM, Mummery WK (2009). Applying GPS to enhance understanding of transport-related physical activity. J Sci Med Sport.

[7] Sallis JF, Prochaska JJ, Taylor WC (2000). A review of correlates of physical activity of children and adolescents. Med Sci Sports Exerc.

[8] Davison KK, Lawson CT (2006). Do attributes in the physical environment influence children's physical activity? A review of the literature. Int J Behav Nutr Phys Act.

[9] Maddison R, Ni Mhurchu C (2009). Global positioning system: a new opportunity in physical activity measurement. Int J Behav Nutr Phys Act.

[10] Sandercock G, Angus C, Barton J (2010). Physical activity levels of children living in different built environments. Prev Med.

[11] Ding D, Sallis JF, Kerr J, Lee S, Rosenberg DE (2011). Neighborhood environment and physical activity among youth a review. Am J Prev Med.

[12] Kaczynski AT, Henderson KA (2007). Environmental correlates of physical activity: A review of evidence about parks and recreation. Leisure Sci.

[13] Lachowycz K, Jones AP (2011). Greenspace and obesity: a systematic review of the evidence. Obes Rev.

[14] Panter JR, Jones AP, van Sluijs EM (2008). Environmental determinants of active travel in youth: a review and framework for future research. Int J Behav Nutr Phys Act.

[15] Reimers AK, Mess F, Bucksch J, Jekauc D, Woll A (2013). Systematic review on measurement properties of questionnaires assessing the neighbourhood environment in the context of youth physical activity behaviour. BMC Public Health.

[16] Krenn PJ, Titze S, Oja P, Jones A, Ogilvie D (2011). Use of global positioning systems to study physical activity and the environment: a systematic review. Am J Prev Med.

[17] Cerin E, Saelens BE, Sallis JF, Frank LD (2006). Neighborhood Environment Walkability Scale: validity and development of a short form. Med Sci Sports Exerc.

[18] Saelens BE, Sallis JF, Frank LD (2003). Environmental correlates of walking and cycling: findings from the transportation, urban design, and planning literatures. Ann Behav Med.

[19] Spittaels H, Verloigne M, Gidlow C, Gloanec J, Titze S, Foster C, Oppert JM, Rutter H, Oja P, Sjostrom M, De Bourdeaudhuij I (2010). Measuring physical activity-related environmental factors: reliability and predictive validity of the European environmental questionnaire ALPHA. Int J Behav Nutr Phys Act.

[20] Adamo KB, Prince SA, Tricco AC, Connor-Gorber S, Tremblay M (2009). A comparison of indirect versus direct measures for assessing physical activity in the pediatric population: a systematic review. Int J Pediatr Obes.

[21] Bedimo-Rung AL, Mowen AJ, Cohen DA (2005). The significance of parks to physical activity and public health: a conceptual model. Am J Prev Med.

[22] Chaix B, Meline J, Duncan S, Merrien C, Karusisi N, Perchoux C, Lewin A, Labadi K, Kestens Y (2013). GPS tracking in neighborhood and health studies: a step forward for environmental exposure assessment, a step backward for causal inference?. Health Place.

[23] Coombes E, van Sluijs E, Jones A (2013). Is environmental setting associated with the intensity and duration of children's physical activity? Findings from the SPEEDY GPS study. Health Place.

[24] Cooper AR, Page AS, Wheeler BW, Griew P, Davis L, Hillsdon M, Jago R (2010). Mapping the walk to school using accelerometry combined with a global positioning system. Am J Prev Med.

[25] Jones AP, Coombes EG, Griffin SJ, van Sluijs EM (2009). Environmental supportiveness for physical activity in English schoolchildren: a study using Global Positioning Systems. Int J Beh Nutr Phys Act.

[26] Lachowycz K, Jones AP, Page AS, Wheeler BW, Cooper AR (2012). What can global positioning systems tell us about the contribution of different types of urban greenspace to children's physical activity?. Health Place.

[27] Mackett R, Brown B, Gong Y, Kitazawa K, Paskins J (2007). Children's Independent Movement in the Local Environment. Built Environ.

[28] Southward EF, Page AS, Wheeler BW, Cooper AR (2012). Contribution of the school journey to daily physical activity in children aged 11–12 years. Am J Prev Med.

[29] Wheeler BW, Cooper AR, Page AS, Jago R (2010). Greenspace and children's physical activity: a GPS/GIS analysis of the PEACH project. Prev Med.

[30] Almanza E, Jerrett M, Dunton G, Seto E, Pentz MA (2012). A study of community design, greenness, and physical activity in children using satellite, GPS and accelerometer data. Health Place.

[31] Oreskovic NM, Blossom J, Field AE, Chiang SR, Winickoff JP, Kleinman RE (2012). Combining global positioning system and accelerometer data to determine the locations of physical activity in children. Geospatial Health.

[32] Rodriguez DA, Cho GH, Elder JP, Conway TL, Evenson KR, Ghosh-Dastidar B, Shay E, Cohen D, Veblen-Mortenson S, Pickrell J, Lytle L (2012). Identifying walking trips from GPS and accelerometer data in adolescent females. J Phys Act Health.

[33] Rodriguez DA, Cho GH, Evenson KR, Conway TL, Cohen D, Ghosh-Dastidar B, Pickrel JL, Veblen-Mortenson S, Lytle LA (2012). Out and about: association of the built environment with physical activity behaviors of adolescent females. Health Place.

[34] Maddison R, Jiang Y, Vander Hoorn S, Exeter D, Mhurchu CN, Dorey E (2010). Describing patterns of physical activity in adolescents using global positioning systems and accelerometry. Pediatr Exerc Sci.

[35] Quigg R, Gray A, Reeder AI, Holt A, Waters DL (2010). Using accelerometers and GPS units to identify the proportion of daily physical activity located in parks with playgrounds in New Zealand children. Prev Med.

[36] Rainham DG, Bates CJ, Blanchard CM, Dummer TJ, Kirk SF, Shearer CL (2012). Spatial classification of youth physical activity patterns. Am J Prev Med.

[37] Rainham D, McDowell I, Krewski D, Sawada M (2010). Conceptualizing the healthscape: contributions of time geography, location technologies and spatial ecology to place and health research. Soc Sci Med.

[38] Bromley C, Rutherford L, Hinchliffe S, Sharp C (2013). Chapter 6 Physical activity. The Scottish Health Survey 2012 edition: volume1, main report A National Statistics Publication for Scotland.

[39] Healy GN, Dunstan DW, Salmon J, Cerin E, Shaw JE, Zimmet PZ, Owen N (2007). Objectively measured light-intensity physical activity is independently associated with 2-h plasma glucose. Diabetes Care.

[40] Groenewegen PP, van den Berg AE, de Vries S, Verheij RA, Vitamin G (2006). Eeffects of green space on health, well-being, and social safety. BMC Public Health.

[41] Muñoz S-A (2009). Children in the Outdoors - A literature review. Book Children in the Outdoors - A literature review.

[42] Mass J, Sterkenburg RP, de Vries SI, Pierik F, Stock C, Ellaway A (2013). Using GPS to Measure the Interaction Between Individuals and their Neighbourhood. Neighbourhood Structure and Health Promotion.

[43] Cho GH, Rodriguez DA, Evenson KR (2011). Identifying walking trips using GPS data. Med Sci Sports Exerc.

[44] Kerr J, Duncan S, Schipperijn J (2011). Using global positioning systems in health research: a practical approach to data collection and processing. Am J Prev Med.

[45] Schipperijn J, Ejstrud B, Troelsen J, Stock C, Ellaway A (2013). GIS: A Spatial Turn in the Health Science?. Neighbourhood Structure and Health Promotion.

[46] Chaix B, Kestens Y, Perchoux C, Karusisi N, Merlo J, Labadi K (2012). An interactive mapping tool to assess individual mobility patterns in neighborhood studies. Am J Prev Med.

[47] Department of Health (2011). Start Active, Stay Active: A Report on Physical Activity from the Four Home Countries' Chief Medical Officers. Start Active, Stay Active: A Report On Physical Activity from the Four Home Countries' Chief Medical Officers.

[48] Owen N, Healy GN, Matthews CE, Dunstan DW (2010). Too much sitting: the population health science of sedentary behavior. Exerc Sport Sci Rev.

[49] Tisdall K, Davis JM, Gallagher M (2009). Researching with Children and Young People. ᅟ.

[50] Collins P, Al-Nakeeb Y, Nevill A, Lyons M (2012). The impact of the built environment on young people's physical activity patterns: a suburban-rural comparison using GPS. Int J Environ Res Public Health.

[51] Fjørtoft I, Kristoffersen B, Sageie J (2009). Children in schoolyards: tracking movement patterns and physical activity in schoolyards using global positioning system and heart rate monitoring. Landscape Urban Plann.

[52] Troped PJ, Wilson JS, Matthews CE, Cromley EK, Melly SJ (2010). The built environment and location-based physical activity. Am J Prev Med.

