# Supplementary material for: Combining GPS, GIS, and accelerometry to explore the physical activity and environment relationship in children and young people – a review
Source: Int J Behav Nutr Phys Act. 2014 Sep 13;11:93. doi: 10.1186/s12966-014-0093-0 (PMC4172984; doi:10.1186/s12966-014-0093-0)
Supplement: Additional file 1: Table S2. — PA and place based variables/measures, and study findings of all included articles. [file 12966_2014_93_MOESM1_ESM.docx]

Additional file 1: Table S2. PA and place based variables/measures, and study findings of all included articles

| **First Author** | **Year** | **PA/GPS details/inclusion criteria** | **Main PA/Environment**  **Outcome Measure(s)** | **Place based measures** | **Environmental/Neighbourhood Variables** | | | | | |
| --- | --- | --- | --- | --- | --- | --- | --- | --- | --- | --- |
| Coombes, E. [23] | 2013 | **PA**   - 5sec epoch - No minimum wear time   **GPS**   - Trackpoint recorded for every change of direction or speed   **Inclusion**  **PA**   - No restrictions - 4 days included in analysis | - % of recorded time children spent in light, moderate, vigorous, bout MVPA, and non-bout MVPA in different land use categories - MVPA bout – at least 5 mins (30% of bout allowed below this intensity) - ≥ 2000 counts per minute (cpm) (Ekelund et al., 2004) | None used | **Time spent in specific land use types**  **GIS data source:**   - Ordnance Survey Mastermap (OSMM) - Centre for Ecology (CEH) Land Cover Map of GB | | | | | |
|  |  |  |  |  | **Land use types:**   - Buildings - Other built land use (e.g. car parks) - Roads & pavements - Domestic gardens - Parks | | | | | - Farmland - Grassland - Woodland - Beaches |
|  |  |  |  |  | **ArcGIS 9.2 (ESRI, Redlands, CA)** | | | | | |
| Almanza, E.  [30] | 2012 | **PA**   - 30sec epoch   **GPS**   - 30sec interval   **Inclusion:**   - ≥ 3 days with 4h of combined data - Home and school-time removed - Data in neighbourhood retained - 500 metre buffer around home - Days with <1hr of neighbourhood data excluded from analysis | **MVPA**   1. Momentary probability – association between 30-sec exposure to greenness and the odds of MVPA 2. Mean daily mins in neighbourhood  - Age-specific Freedson cut-points (2005) | **Home**  30m Euclidean distance buffer  **Neighbourhood**  500m radius around the home | **Greenness**   - Normalized Difference Vegetation Index (NDVI) per GPS point - Low (-1) to High(+1) vegetation   **GIS data source:**   - Landsat5 Thematic Mapper satellite imagery (US Geological Survey)   **Greenness variables**   1. NVDI index per 30 sec epoch 2. **Categorical variable**:   Nearly 0, 1.5-20, or >20min/day exposure to greener spaces (greener = > 90^th^ percentile of all points in analysis)   1. **Mean NDVI value for neighbourhood**   **ArcGIS 9.3 (ESRI, Redlands, CA)** | | | | | |
| Lachowycz, K. [26] | 2012 | **PA**   - 10sec epoch - 7 days recording   **GPS**   - 10sec epoch - Worn for 4 days - end of school until bedtime   **Inclusion**   - Accelerometer data and at least 1 min of GPS combined. - Not clear if all 4 days were needed to be included in analysis | **MVPA**   - cpm were re-calculated per 10 sec epoch - Expressed as mean minutes and % of total time - >3200 cpm (Puyau, et al., 2002) | **General**  GPS only considered if it fell within the boundaries of Bristol Local Authority | **Greenspace**  **GIS data source:**   - Ordnance Survey Mastermap (OSMM) Topography - Bristol City Council (parks data) | | | | | |
|  |  |  |  |  | **Land use categories:**  **Greenspace (overall)**   - Parks - Private gardens - School grounds - Other greenspace | | | **Other land use**   - Roads/pavements - Green verges - Built surfaces | | |
|  |  |  |  |  | **ArcGIS 9 (ESRI, Redlands, CA)** | | | | | |
| Oreskovic, N. [31] | 2012 | **PA**   - 30sec epoch (collapsed to 1min for analysis) - Waking hours for 7 days   **GPS**   - Trackpoint every 3 to 55secs - 1^st^ GPS point of every minute matched with PA - Waking hours for 7 days   **Inclusion**   - At least 1 full hour of matched data recorded on at least 1 of the 7 days | **PA intensity**   - Freedson age-specific PA intensity cut-points - MVPA used for paper - >1952 cpm   **Additional variables**   - Total PA/day - Daily MVPA - Time spent in each location - Total time spent in all intensity levels (light, moderate, vigorous) in each location | **Home**   - Home postcodes geocoded - All GPS points within 25m of centre of the home were classified as ‘home’   **School**   - Position geocoded - All GPS points falling within 100m of school perimeter classified as ‘school’ | **Land use**  **GIS data source:**   - Commonwealth of Massachusetts’ Office of Geographic Information (**MassGIS**) digital orthoimagery for Revere - Detailed colour imagery – accuracy of 3m   **Child location categories:**   - Home - School - Car - Indoor/other (non-home, non-school) - Park/play-ground (all outdoor recreation spaces) - Street/walking   Areas with GPS points visually scanned at 1:4,000 scale and land use locations interpreted based on photo interpretation.  **ArcGIS 9.2 (ESRI, Redlands, CA)** | | | | | |
| Rainham, D. [36] | 2012 | **PA**   - 30sec epoch - 8 days recording   **GPS**   - 1sec epoch - 8 days recording   **Inclusion**  **PA**   - ≥ 8 hrs of activity   **Combined**   - At least 3 days of corresponding GPS/accelerometer data - > 10 hours/day | **MVPA**   - Mins of MVPA - % of time in MVPA for each category by gender and urbanicity - Average mins of MVPA by urbanicity, school SES, home, school, commuting, and all other locations combined   **Authors did not present how MVPA was classified** | None used | **Location of activity**  **GIS data source:**   - Street network - Municipal cadastral data - Satellite imagery - Enhanced points-of-interest file (TeleAtlas) | | | | | |
|  |  |  |  |  | **Location Category:**   - Home - School - Commuting - Other Locations - Athletic facility - Entertainment - Greenspace - Military | | - Parking lot - Religious - Residential - Restaurant - Retail - Services - Transportation | | | |
| Rodriguez, D. [33] | 2012 | **PA**   - 30sec epoch - Aggregated to 1min for analysis - Waking hours 6 days   **GPS**   - 1min interval   **Inclusion**  **PA**   - Weekday valid time > 10.6hrs - Weekend valid time > 8.3hrs   **Combined**   - At least 1 GPS point/day - 1 day of valid hours required for inclusion | **PA intensity**   - Minute by minute intensity - Treuth et al. (2004) threshold intensity classification for adolescent girls used   **Sedentary -**<100cpm  **Light -** ≥100 - <3000cpm  **MVPA -** ≥3000cpm | **Environment**  50m circle around each GPS/accelerometer point  **Neighbourhood**  800m buffer around home  Points falling within 60 metre buffer around home and school excluded | **GIS data source:**   - US Census Bureau - Census Transportation Planning Package - Neighbourhood Impact on Kids study - City and county health department inspection records | | | | | |
|  |  |  |  |  | **Built environment (50m buffer)**   - Street density - No. of food outlets - Presence of parks - Presence of schools - Presence of PA facilities - Presence of fast food rest   **ArcGIS 9.2 (ESRI, Redlands, CA)** | **Home neighbourhood (800m buffer)**   - Gross population density - Ratio between jobs and households - Distance to nearest park (miles) - Distance to own school - Intersection density - Road density - % households under level of   poverty | | | | |
| Rodriguez, D. [32] | 2012 | **PA**   - 30-sec epoch - 1min for analysis - Waking hours for 6 days   **GPS**   - 1min interval - Waking hours for 6 days   **Inclusion**  **PA**   - Wkday > 10.6hrs/day - Wkend > 8.3hrs/day   **Combined**   - at least 1 GPS point/day - 1 day of valid hours - School data excluded | **Location of destinations post walking trip**  **Walking trips**   1. Accelerometer/GPS 2. Diary   **1.Accelerometer/GPS**   - PA counts - Bout length - Speed (GPS) - Dwell time (GPS)   **2.Diary**  The Neighbourhood Places Log (NPL)   - Travel mode - Destination name, address, arrival and departure time | None used | - 0.4km circle drawn around the final GPS point of a trip - All street names fully or partially contained in the circle identified - Using ESRI Streetmap in ArcCatalog (ESRI, Redlands, CA) - Street address associated with each diary entered destination compared to the GIS   **ArcMap 9.2 (ESRI, Redlands, CA)** | | | | | |
| Southward, E. [28] | 2012 | **PA**   - 10sec epoch   **GPS**   - 10sec interval - 3 days recording   **Diary**  Travel mode  **Inclusion**  **PA & GPS Combined**   - Walked to school - provided data from at least one **journey** both to and from school (8am and 9am   3pm and 5pm) | **MVPA**   - Mins of MVPA/day - Mins of MVPA on school journey - ≥2296 cpm threshold used (Evenson, 2008) | **Home**  Postcodes geocoded and mapped  **School**  Addresses geocoded and mapped | - Journeys to and from school mapped in GIS   **ArcGIS 9.3 (ESRI, Redlands, CA)** | | | | | |
| Cooper, A.  [24] | 2010 | **PA**   - 7 days wear time - 10sec epoch   **GPS**   - 10sec epoch - 2 days collection   **Inclusion**  **PA**   - ≥ 2 days with ≥ 480 mins/day (7am-11pm)   **PA & GPS Combined**   - Walked to school - provided data between 8am and 9am | **MVPA/day**   - Mean CPM   Minutes of MVPA/hr &/day   - ≥10 mins zero counts excluded - MVPA = >3200 cpm (Puyau, et al., 2002) recording | **General**  School journey analysis therefore only GPS/PA data from those who walked to school were analysed.  No neighbourhood component | **Polygon around school playground**   - Data falling inside polygon - **Playground** - Data falling outside polygon - **Journey**   **GIS ArcMap 9.2** | | | | | |
|  |  |  |  |  |  |  |  |  |  |  |
|  |  |  |  |  |  |  |  |  |  |  |
|  |  |  |  |  |  |  |  |  |  |  |
|  |  |  |  |  |  |  |  |  |  |  |
| Maddison, R. [34] | 2010 | **PA**   - 1min epoch - 4 consecutive days (waking hours)   **GPS**   - Recorded trackpoints on change of direction or speed - 4 consecutive days (waking hours)   **Inclusion**  **PA**   - ≥ 10 hrs/day - At least 3 valid days (including 1 weekend day) | **PA intensity**   - Mean time/day - Total time and % of total time (all 4days) spent in light, moderate, vigorous and MVPA - Weekday - Weekend - School day (0900-1530) - Bouts of MVPA - ≥ 10 consecutive mins - 30% tolerance - Freedson, age specific, cut points used (Freedson (2009) | **Home**  Address geocoded using GeoStan Map (Critchlow Associates, Wellington, NZ)  150m Euclidean buffer  **School**  1km Euclidean buffer placed around school | **GIS data source:**   - Road network - Land use - Building footprints   **ArcGIS 9.3.1. (ESRI, Redlands, CA)** | | | | | |
| Wheeler, B.  [29] | 2010 | **PA**   - 10 sec epoch - 7 days recording   **GPS**   - 10 sec epoch - Worn for 4 days after school (until bedtime) - 1 weekend day (08:00-22:00)   **Inclusion**   - Accelerometer data and at least 1 min of GPS combined. - Not clear if all 4 days were included in analysis | **MVPA**   - >3200 cpm (Puyau et al., 2002) - cpm were re-calculated per 10 sec epoch | **General**  Land use and topography data was available for 40km^2^ of the study area. 2% of the GPS points fell out with this area and subsequently removed from the analysis. | **Greenspace**  **GIS data source:**   - Generalised Land Use Database (GLUD) - Ordnance Survey Mastermap (OSMM) Topography   Used to tell if each data point occurred within or out with GS.  **ArcGIS 9 (ESRI, Redlands, CA)** | | | | | |
| Quigg, R.  [35] | 2010 | **PA**   - 3 second epoch - 1 min for analysis - 6 consecutive days   **GPS**   - 1 min epoch   **Inclusion**  **PA**   - 5hrs of data needed/day   **GPS**   - None reported   **Combined**   - At least 1 day matched | Proportion of daily activity located in city parks with playgrounds   - **Total counts/day** - **% total counts spent in parks** | **General**  Home locations geocoded using home address | **GIS data source:**   - Park cadastral boundaries provided by Dunedin City Council - Regional parks of Dunedin hinterland excluded – based on definition of parks having playgrounds   **ArcGIS 9.2 (ESRI, Redlands, CA)** | | | | | |
| Jones, A.  [25] | 2009 | **PA**   - 5 sec epoch - 4 consecutive days (waking hrs)   **GPS**   - Recorded with change of direction or speed (1 to 10 secs) - 4 consecutive days (waking hrs)   **Inclusion**  **PA**   - No minimum wear time (prevalence not the study focus) | **MVPA**   - MVPA bout – at least 5 mins (30% of bout allowed below this intensity) - ≥ 2000 cpm (Ekelund et al., 2004) | **Neighbourhood**  800m surrounding address along pedestrian network (roads & pub footpaths)  Home locations via postcode  Ordnance Survey Address Point database | **Time spent in specific land use types**  **GIS data source:**   - Ordnance Survey Mastermap (OSMM) - Centre for Ecology (CEH) Land Cover Map of GB | | | | | |
|  |  |  |  |  | **Land use types:**   - Buildings - Other built land use - Roads & pavements - Gardens - Parks   **ArcGIS 9.2 (ESRI, Redlands, CA)** | | | | - Farmland - Grassland - Woodland - Beaches | |
| Mackett, R.  [27] | 2007 | **PA**   - 1min epoch - Vector magnitude - 4 consecutive days   **Activity diary**  domains of activity:   - Walking, Playing, Organised clubs   **GPS**   - Garmin Foretrex 201 - 4 consecutive days   **Inclusion**   - None stated   **Exclusion**   - containing car trips only - outdoor GPS <5mins | - Speed - metres/sec - Intensity (10^-2^ activity calories/ min) - Angle   Each presented for:   - Gender - Road v open space - walking activity/ playing/ organised clubs | None used | **GIS data source:**   - OSMM topology layer   **Land use types:**   - Roads, tracks and paths - Other space (public open space)   GPS points on private space (e.g. gardens attached to house) removed | | | | | |
