# Supplementary material for: Combining GPS, GIS, and accelerometry to explore the physical activity and environment relationship in children and young people – a review
Source: Int J Behav Nutr Phys Act. 2014 Sep 13;11:93. doi: 10.1186/s12966-014-0093-0 (PMC4172984; doi:10.1186/s12966-014-0093-0)
Supplement: Additional file 2: Table S3. — Research questions and main findings of included articles. [file 12966_2014_93_MOESM2_ESM.doc]

Additional file 2: Table S3. Research questions and main findings of included articles

| First Author [reference] |  | Findings |  | |  | |
| --- | --- | --- | --- | --- | --- | --- |
| Coombes, E. [23] | Research Question | Descriptive | ANOVA | | t-test | |
|  | Is the environmental setting associated with the intensity and duration of children’s physical activity? | • Boys spend more time in all intensities than females | **•** 24% of light (L) intensity activity occurred in buildings | | Non bout MVPA significantly > MVPA bout time in: | |
|  | Analysis | • Both boys and girls spent approximately 60% of recorded time in gardens and buildings. | • Moderate (M, 20%), vigorous (V, 18%) – difference (p<0.001, L > M & V) | | • Buildings - 22% vs 7%, p<0.001 (5mins more/day) | |
|  | ANOVA |  |  | | • Other built land - 16% vs 11%, p=0.015 (3mins more/day) | |
|  | % of light, moderate, and vigorous activity undertaken in each land use |  |  | | • Domestic gardens - 29% vs 21%, p<0.001 (6min more/day) | |
|  | t-test |  | **•** 13% of light intensity activity occurred on roads & pavements | | Bout MVPA significantly > than non-bout MVPA in: | |
|  | % time spent in bout MVPA v non-bout MVPA; compared for each land use type |  | • moderate (12%) and vigorous (9 %) – difference (p<0.001, L> M & V) | | • Roads & pavements - 17% vs 9%, p<0.001 (36sec more/day) | |
|  |  |  | • 31% of vigorous intensity activity occurred in domestic gardens | | • Urban/rural and gender stratifications did not substantially modify the results. | |
|  | Stratified analyses by gender and urban/rural status |  | • light (29%) and moderate (27%) - difference (p=0.009, V > L & M) | |  |  |
|  |  |  | • Actual time = 4mins/day in vigorous, 26mins/day in light, and 7mins/day in moderate | |  |  |
|  |  |  | • Significantly greater % of vigorous undertaken in parks (p=0.01) and grassland (p=0.005) compared to light and moderate. Equated to <1.5 min per day in each land use | |  |  |
| Almanza, E. [30] | Aim | Descriptive (SGC vs CC) | Logistic regression | | Multiplicative indicator on MVPA of greenness exposure | |
|  | Association between greenness and MVPA across smart growth community (SGC) and conventional community (CC) | Time spent in neighbourhood (Mdn) | Exposure to greenness and odds of MVPA (sedentary ref group): | | • 1.5 - 20 mins exposure to greenness resulted in 2.11 times more MVPA than those with nearly zero exposure | |
|  |  | - 51.64 mins vs 31.86 mins (p=0.01) |  |  |  |  |
|  | Smart growth = mixed land use; diverse housing and transportation options; connected, walkable streets; areas for social interaction; compact building design | MVPA in neighbourhood (Mdn) | • 1.34 for conventional area | | • >20 mins greenness – 4.72 times the rate of MVPA compared to nearly zero greenness | |
|  |  | 7.5 mins vs 4.25 mins (p=0.05) | • 1.39 for smart growth area | | • Mean neighbourhood NDVI not associated with daily neighbourhood-MVPA | |
|  |  | Neighbourhood NDVI (mean) |  |  |  |  |
|  |  | 0.10 vs 0.05 (p<0.001) |  |  |  |  |
|  | Analysis |  |  |  |  |  |
|  | Logistic regression |  |  |  |  |  |
|  | • Association of epoch level greenness and odds of MVPA |  |  |  |  |  |
|  | • Greenness measured through the Normalized Difference Vegetation Index (NDVI) |  |  |  |  |  |
|  | Negative binomial generalized linear modelling |  |  |  |  |  |
|  | • Multiplicative indicator on MVPA of greenness exposure |  |  |  |  |  |
|  | Gender, age, income, race, and BMI entered into model |  |  |  |  |  |
| Lachowycz, K. [26] | Aim | Descriptive | Weekday v weekend (% outdoor MVPA time) in: | Saturday v Sunday (% outdoor MVPA time) | Seasons (% outdoor MVPA time) | |
|  | To investigate the contribution of different types of urban greenspace to children’s physical activity | Outdoor MVPA (% of total MVPA) | Greenspace (overall) | • % MVPA time greater on Sunday (p<0.001) | No significant seasonal differences for: | |
|  |  | • Weekday evenings (26.4%) | • greater in weekend than weekday |  | Total time in MVPA, MVPA time outdoors, MVPA time in all greenspace, MVPA time in park sub-types. | |
|  |  | • Weekend (17.6%) | (46% vs 34% p<0.001) |  |  |  |
|  | Analysis | Outdoor MVPA time spent in greenspace/day (% of total daily outdoor MVPA) | All park types (except sports area) |  | % of total MVPA - Weekday | |
|  | • Group difference tests (specific tests not stated) | • 2.4mins (33.6%) / 3.5mins (46.0%) | • greater in weekend than weekday |  | • % outdoor MVPA in parks lower in winter (7%) and spring (7.7%) than summer (17.2%) and autumn (11.2%) (p<0.001). | |
|  | • Weekday evening (15:00-22:00) vs weekend (08:00-22:00) | (weekday evening and weekend day respectively) | (29% vs 10%, p<0.001) |  |  | |
|  | • Saturday vs Sunday |  | Private garden |  | % of total MVPA - Weekend | |
|  | • Seasons (Spring/Summer/Autumn/Winter) |  | • greater during weekday than weekend |  | • % outdoor MVPA highest in winter (12.2%) and lowest in summer (3.2%) (p<0.001). | |
|  |  |  | (22% vs 16.1%, p<0.001) |  | No differences found in % of outdoor MVPA occurring in greenspace overall or parks. | |
| Oreskovic, N. [31] | Aim | Descriptive | MVPA (mean) | Winter (% of total MVPA) | Spring (% of total MVPA) | Summer (% of total MVPA) |
|  | Assessment of the locations of activity and intensity of PA within each location | • Most of time spent at home (vs other land use) | • 15.4mins/day across whole period | • 43% at home | • 44% on the streets/walking | • 57% in parks/playgrounds |
|  | No statistical tests conducted | • Consistent across seasons | • 1.7mins/day in winter | • 29% on streets/walking | • 34% at home | • 12% at home |
|  | Descriptive data only | • 76% in winter, 43% in spring, and 42% in summer | • 36.4mins/day in spring | • 15% indoors/other | • 9% at school | • 11% on streets/walking |
|  |  |  | • 6.3mins/day in summer | • 12% at school | • 9% in parks/playgrounds | • 10% indoors/other |
| Rainham, D. [36] | Aim | Descriptive | % of total MVPA time attributed to specific locations | | MVPA time - Urbanicity and SES (by location) - Kruskall-Wallis H test | |
|  | To investigate the MVPA differences at locations by urbanicity and SES. | Girls had more mins of MVPA than boys | • URBAN | | • No differences found in MVPA across urbanicity at home. | |
|  |  | • (129.4 ± 153.8 vs 101.6 ± 103.3). | • Commuting (55.5% girls, 57.6% boys) | | • Urban students achieved more MVPA than suburban and rural at school. | |
|  |  |  | • Home and school (34.4/% girls, 33.6% boys) | | • (45.7 vs 18.6 vs 29.8mins, p<0.001). | |
|  |  |  | • Greenspace (1.3% girls, 0.6% boys) | |  |  |
|  | Analysis | MVPA decreased with age: | SUBURBAN | | • Urban students achieved more MVPA than suburban and rural during **commuting**. | |
|  | • Kruskall-Wallis H test – group differences by location | • 12-yrs – 167.9 ± 148.6mins | • Home and school (41.8% girls, 52.7% boys) | | • (110.3 vs 31.5 vs 19.5mins, P<0.001). | |
|  |  | • 16-yrs – 30.3 ± 39.3mins | • Commuting (42.5% girls, 27.4% boys) | |  | |
|  | • Groups | Urban student’s greater total MVPA than suburban and rural students. | • Greenspace (2.5% girls, 3.9% boys) | | • Urban students achieved more MVPA than suburban and rural in **other** locations. | |
|  | • Urbanicity | • Urban - 196.6 ± 163.8 mins | • RURAL | | • (19.7 vs 14.8 vs 12.0mins, p=0.03). | |
|  | • SES | • Suburban - 84.9 ± 103.2 mins | • Home and school (65% girls, 58.3% boys) | | • No SES differences by location | |
|  | • Location | • Rural - 81.7 ± 98.2 mins | • Commuting (20.7% girls, 27% boys) | |  |  |
|  | • Home | • p<0.05 | • Greenspace (4.8% girls, 5.6% boys) | |  |  |
|  | • School |  |  |  |  |  |
|  | • Commute |  |  |  |  |  |
|  | • Other |  |  |  |  |  |
| Rodriguez, D. [33] | Aim | | San Diego | | Minneapolis | |
|  | To investigate the association of the built environment with physical activity behaviours of adolescent females | | Ref category - Sedentary | | Ref category -Sedentary | |
|  |  | | Presence of parks | | Presence of parks | |
|  | Analysis | | OR 1.41 (41% increased odds of light intensity) | | OR 1.86 (MVPA) | |
|  | Random intercept models using the generalized linear latent and mixed models (GLLAMM) extension with a multinomial logit link function, a binomial family distribution, and the adaptive quadrature. | | Higher pop density (1000s/sq mile) | | Higher pop density | |
|  |  |  | OR 1.01 (MVPA) | | OR 1.04 (MVPA) | |
|  | • Unit of analysis - GPS/accelerometer point (multiple points/participant) | | Presence of schools | | Presence of schools | |
|  |  | | OR 1.69 (MVPA) | | OR 2.14 (MVPA) | |
|  | Adjusted for home neighbourhood variables, the fixed effects of the measurement time period (1^st^ or 2^nd^), and whether the point was from the weekend or weekday. | | Road length (miles) | | Road length | |
|  |  |  | OR 0.38 (MVPA) | | OR 0.43 (MVPA) | |
|  |  |  | No. food outlets | | No. food outlets | |
|  |  |  | OR 0.73 (MVPA) | | OR 0.71 (MVPA) | |
|  |  |  | San Diego (weekend) | | Minneapolis (weekend) | |
|  |  |  | OR 0.87 (Light) | | OR 0.62 (MVPA) | |
|  |  |  | OR 0.39 (MVPA) | | Activity at 2^nd^ year measurement | |
|  |  |  |  |  | 0.54 (Light) | |
|  |  |  |  |  | 0.51 (MVPA) | |
|  |  |  | Adjusted odds ratio of minute-by-minute physical activity intensity (sed ref category vs either light or MVPA) associated with the built environment around each point | | Adjusted odds ratio of minute-by-minute physical activity intensity (sed ref category vs either light or MVPA) associated with the built environment around each point | |
| Rodriguez, D. [32] | Aim | | Descriptive | | Location agreement | |
|  | To investigate the agreement of ‘locations visited’ by diary and accelerometer/GPS (GIS measured) | | • Average of 0.54 walking trips/day | | **Calibration Sample** | |
|  |  |  | • 0 walking trips reported on 122 person-days (67.4%) | | Between 91% and 100% of locations matched between GPS and diary. | |
|  |  |  | • 4 trips reported on two person-days (1.1%) | | **Validation Sample** | |
|  | The paper tested multiple algorithms, made up from varying parameters, to identify walking trips in a calibration and validation sample. These were compared between accelerometry/GPS and diary entries | |  | | Between 86% and 88% of locations matched between GPS and diary. | |
| Southward, E. [28] | Aim | | Contribution of Journeys to PA - Paired sample t-test | | Independent samples t-test | |
|  | To investigate the contribution that the journey to and from school makes to PA levels | | • No difference in MVPA between journey to (11 out of 20mins) or from (12 out of 23mins) school - Approximately 50% of both journeys were MVPA. | | • No difference in PA by gender to school - Boys (13.9mins) vs Girls (14.8mins) | |
|  |  |  | • Each journey contributed between 16%-18% of daily MVPA. | |  |  |
|  | Analysis | |  |  | • No difference in PA by gender from school | |
|  | Paired sample t-test | |  |  |  |  |
|  | • Journey to school v journey from school | |  |  | Boys (23.2mins) vs Girls (20.7mins) | |
|  |  | |  |  | • Boys had significantly more daily MVPA than girls | |
|  |  |  |  |  | Boys (73.2mins) vs Girls (61.5mins), p = 0.007 | |
|  | Independent samples t-test | |  |  | • Girl’s journey to and from school contributed more MVPA/day | |
|  | • Boys v girls | |  |  | Girls (35.6%) vs Boys (31.3%); no p value stated | |
|  | • Distance to school | |  |  |  | |
|  | <3km and >3km (round trip) | |  |  | • Total journey MVPA increased as distance from school increased (p for trend = <0.001) | |
|  |  |  |  |  | • Those with round trip of >3km had higher overall MVPA/day - >3km (73.8mins) v <3km (60.2mins); p<0,001) | |
|  |  |  |  |  | • Journey to school contributed greater proportion of MVPA/day in >3km group compared to <3km group - (39% vs 28.6%). | |
| Cooper, A. [24] | Aim | | Descriptive | Active travel and overall PA | Active travel and overall PA | Contribution of ‘journey to school’ to PA/MVPA before school – Mean values (SD) |
|  | To investigate the contribution of travel mode and overall PA/ MVPA levels per day. | | • 51% walked to school (n=70) | 07:00 - 23:00 – Mean values | 08:00 - 09:00 – Mean values | • Journey – 2131.3 cpm (± 1170.7) vs. Playground – 1089.7 cpm (± 938.6) (p<.001) |
|  |  |  | • 34% by car (n = 47) |  |  |  |
|  |  |  | • 13% by bus (n = 18) | Counts per min (CPM **± SD**) | CPM (±**SD)** |  |
|  |  |  | • 2% by bicycle (n = 2) | Walk - 606 ± 162 | Walk - 878.8 ± 387.6 |  |
|  | To investigate the contribution of active travel to activity level and MVPA before school | |  | Car - 530 ± 143.5 | Car - 608.7 ± 264.1 | • 45.6% (5.2 mins) of matched GPS points recorded between 08:00-09:00 were in the journey. |
|  |  | |  | Bus - 556.7 ± 153 | Bus - 747.6 ± 333.9 | • 1.6 mins MVPA in journey |
|  |  | |  | (p=0.011 Walk > Car travel) | (p<0.001 walk >car travel) |  |
|  | Analysis | |  | MVPA | MVPA | • 0.6 mins MVPA in playground |
|  | Active travel and overall PA | |  | Walk – 42.5 ± 17.7 | Walk - 5.4 ± 4.3 |  |
|  | • ANOVA (Walk/Car/Bus) | |  | Car – 38.3 ± 17.2 | Car - 3.3 ± 2.9 | Linear distance between home and school correlated with amount of GPS time recorded on the journey (r=0.495, p<0.001). |
|  | Contribution of ‘journey to school’ to PA/MVPA before school | |  | Bus – 40.2 ± 16.6 | Bus - 5.9 ± 3.7 |  |
|  | • Paired t-test | |  | (n.s all comparisons) | (p < 0.001 Walk > Car travel) |  |
|  |  |  |  | (p<0.05 Bus > Car travel) |  |  |
| Maddison, R. [34] | Aim | | Descriptive | Total time (%) across 4 days – Weekdays | Total time (%) across 4 days - Weekend days | MVPA bout analysis |
|  | Describe the location and intensity of free-living PA at school and home environments | | • Mean MVPA/day | • Total time spent within 1km radius of school **-** 510hrs | • Total time spent within 1km radius of school **-** 55hrs | • 395 bouts of moderate or vigorous PA (87% moderate). |
|  |  |  | = 74mins |  |  |  |
|  |  | | • Mean Vigorous PA/day | • Total time spent within 1km radius of school in MVPA - 84hrs (16%) | • Total time spent within 1km radius of school in MVPA. - 6hrs (11%) | • 325 bouts were on a weekday (90% of all bouts). |
|  |  |  | = 7.5mins |  |  |  |
|  |  |  |  | • 65hrs during school time |  |  |
|  | Analysis | |  | • Total time spent within 150m radius of home - 268hrs | • Total time spent within 150m radius of home - 30hrs | • 71% were within school buffer, 46% were within home buffer. |
|  | • Time spent in MVPA during both week and weekend days | |  | • Total time spent within 150m radius of home in MVPA - 38hrs (14%) | • Total time spent within 150m radius of home in MVPA. - 3hrs (12%) | • Overlap of bout data |
|  | • MVPA bout analysis (10 min or more) | |  |  |  |  |
|  | • No statistical testing conducted | |  |  |  |  |
| Wheeler, B. [29] | Aim | | Descriptive | Logistic Regression | Decreased odds of MVPA (after adjustment) | |
|  | To investigate the likelihood of greenspace and non-greenspace use being of MVPA | | • Only 13% of GPS time was spent outdoors | Boys (MVPA) - Adjusted | • With increasing BMI (both boys and girls) | |
|  |  |  | • Mean counts/epoch (CPE) greater for time outdoors than indoor time for boys and girls (p<0.01) | Adj OR – 4.22 (non-GS outdoor vs indoor) | • In less deprived neighbourhoods (girls) | |
|  |  |  |  | Adj OR – 5.77 (GS outdoor vs indoor) | • During longer daylight hours (boys) | |
|  |  |  |  | Adj OR – 1.37 (GS vs non-GS) | • During summer (girls) | |
|  | Analysis | | • Boys – CPE greater in GS than non-GS (p<0.01) | Girls (MVPA) - Adjusted |  |  |
|  | Logistic regression | | • Girls – no difference between GS and non-GS (p=0.50) | Adj OR – 4.75 (non-GS outdoor vs indoor) |  |  |
|  | Relative odds of an epoch exceeding the MVPA threshold in the different land use types (GS or non-GS), compared to time spent indoors | |  | Adj OR – 5.12 (GS outdoor v indoor) |  |  |
|  | Analysis stratified by gender and adjusted for: BMI, pubertal stage, area deprivation, season (coded as summer or winter), minutes of daylight. | |  | Adj OR – 1.08 (GS vs non-GS) |  |  |
| Quigg, R. [35] | Aim | Descriptive | | Linear regression | | |
|  | Identification of total daily physical activity (TDPA) occurring in city parks | 1.9% of TDPA (1.4 - 2.4, 95%CI) located within a city park with a playground. | | • Higher % of time spent in parks in those classified as obese (2.7%) vs normal (2.0%) and overweight (1.1%) (p=0.023). | | |
|  | Analysis |  |  | • Higher % of TDPA spent in parks for boys than girls (2.4% vs 1.5%, p=0.036). | | |
|  | Mean proportion of the total accelerometer recorded activity that occurred in parks and playgrounds estimated with 95% CI’s |  |  | • Very little activity occurs in parks on schools days after 3pm (0.5%), but significantly different across ages (0.7% vs 0.6% vs 0.2%; 7-8yrs > 9-10yrs and 5-6yrs respectively, p=0.024). | | |
|  | Linear regression |  |  | • On non-school days, boys (2.4 vs 1.5%, boys and girls, p=0.036) and those classified as obese (2.7 vs 2.0 vs 1.1%, obese, normal, and overweight; p=0.023) were more active in city parks. | | |
|  | Used to compare differences in mean proportions across age, BMI and sex categories – adjusting for clustering by schools. |  |  |  | | |
| Jones, A. [25] | Aim | Descriptive | More or less time outdoors (children above or below mean time spent outdoors) | MVPA occurring inside and outside neighbourhood | MVPA in land use type | |
|  | To investigate the relationship between bouts of MVPA and being indoors or outdoors, inside and outside the residing neighbourhood, and land use categories | • Mean MVPA/day (62mins) and bout (15mins) MVPA/day. | • More time outside = more MVPA (45.1mins vs 27.7 mins; outdoors and indoors, p=0.002). | • Mean time spent in activity bouts inside, and outside, the neighbourhood was significantly different for boys and girls (p = 0.05), and urban and rural children (p = 0.01). | • Gardens and street environment (roads and pavements) most used land use types. | |
|  | Analysis | • 62.5% of all activity bouts inside own neighbourhoods. | • Held true regardless of sex and urban/rural status (p<0.05). | • Boys (vs girls, 39.6% vs 33%) and rural (vs urban, 42.8% vs 35%) children engaged in higher proportions of MVPA outside the neighbourhood. | • In rural areas boys use farm and grassland whilst girls more active in built environment (interaction effect p <0.001). | |
|  | Bout MVPA differences in those above and below mean time spent outdoors |  |  |  |  | |
|  | • Independent samples t-tests |  |  |  |  | |
|  | MVPA occurring inside, and outside, neighbourhood |  | • More pronounced for rural (58.0 vs 17.1 mins) and girls (28.8mins vs 17.0 mins). | • Interaction effect - Urban boys more likely than girls to undertake bouts outside neighbourhood (p<0.01). | • Urban gardens and greenspace important. Safe street environments maintained. | |
|  | • Stratified by gender and urban/rural status |  |  |  |  |  |
|  | • Pearson Chi-squared tests |  |  |  |  |  |
|  | MVPA in land use type |  |  |  |  | |
|  | • Stratified by gender and urban/rural status |  |  |  |  | |
|  | • Pearson Chi-squared tests |  |  |  |  | |
| Mackett, R. [27] | Aim | Gender | Land use | | Activity categories | |
|  | GPS used to match with activity diary and extract time spent walking, playing and in organised clubs. Outcome measures compared across each category | • Boys walked faster than girls (0.8m/s vs 0.6 m/s; p=0.029). | • Speed was higher on Roads v Open space (0.8 vs 0.7 m/s; p<0.001). | | • Walking activities (against Playing and Clubs) had the fasted speed associated with them (0.8 v 0.3 v 0.5 m/s; p<0.005). | |
|  | Analysis | • Boys used more energy walking than girls (0.75 vs 0.59 activity cals/min; p=0.001). | • Intensity was higher on Roads v Open space (0.67 vs 0.58 activity cals/min; p<0.001). | | • Walking activities (against Playing and Clubs) had the greatest intensity associated with them (0.65 v 0.52 v 0.41 activity cals/min; p<0.005). | |
|  | Independent t-tests |  |  | | | |
|  | Gender |  |  | | | |
|  | Land use |  |  | | | |
|  | ANOVA | • Presented for unaccompanied walking (without an adult) |  | | | |
|  | Category of activity domain |  |  | | | |
